# Abdominal aortic aneurysm rupture presenting with focal weakness and altered mental status: a case report

**DOI:** 10.1186/s12245-022-00433-5

**Published:** 2022-06-22

**Authors:** Brigid M. Garrity, Eric Sugarman, Stephen Pulley

**Affiliations:** 1 Philadelphia College of Osteopathic Medicine, 4170 City Ave, PhiladelphiaPhiladelphia, PA 19131 USA; 2 Suburban Community Hospital, Norristown, PA 19401 USA

**Keywords:** Abdominal aortic aneurysm rupture, Altered mental status, Cerebrovascular attack, Embolic stroke

## Abstract

**Background:**

Abdominal aortic aneurysms (AAA) can present asymptomatically and may be found through routine screening or seen incidentally on imaging. Rupture due to weaking of the aortic wall is the main complication of an AAA and leads to approximately 200,000 deaths annually worldwide. Clinically, AAA rupture most frequently presents with abdominal and/or back pain, pulsatile abdominal mass, and hypotension. Here, we present an unusual presentation of embolic cerebrovascular accident associated with an AAA rupture.

**Case presentation:**

A 58-year-old African American man transported to the emergency department via ambulance presents with altered mental status and unilateral extremity weakness. The initial presentation was concerning for acute cerebrovascular accident, acute kidney injury, severe sepsis, and urinary tract infection. Several hours after the initial presentation, the patient’s abdomen began to appear distended and he became hypotensive. An abdominal CT was ordered which showed a large AAA rupture with a retroperitoneal bleed. The patient was transferred to a higher-level medical center for surgical repair.

**Conclusion:**

Abdominal aortic aneurysm rupture can rarely present due to an acute cerebrovascular accident with altered mental status and focal neurologic deficits.

## Background

Patients with cerebrovascular accidents (CVAs), intracranial hemorrhages, and other acute neurologic pathologies often present with altered mental status (AMS), neurologic deficits, and hypertension [1]. Patients with a ruptured abdominal aortic aneurysm (AAA) typically present with abdominal, back or flank pain, pulsatile abdominal mass, and hypotension [2, 3]. AAAs occur due to progressive weakening of the aortic wall. Rupture can cause the accumulation of the blood in the abdomen and/or retroperitoneal area leading to shock and possibly death. Many patients die before they present to a healthcare facility, with approximately 200,000 deaths reportedly annually worldwide [3]. We report a case of an AAA rupture in a patient that presented with AMS, facial asymmetry, and a NIH stroke scale of 20, with a benign initial abdominal exam. The purpose of this case is to remind physicians that stroke-like symptoms can rarely be signs of non-neurologic life-threatening conditions like an AAA rupture. Therefore, after an initial stroke work-up, patients should also be evaluated for other serious conditions that may have initially been lower on the differential.

## Case presentation

An initially unknown “John Doe” male presented to the emergency department (ED) via emergency medical services (EMS) with concern for stroke. He lived in a boarding home and was found unresponsive in a common bathroom by new housemates who called 911. They reported that the patient appeared well when he came home from work. About 6 hours later, they found the bathroom door locked and pried it open to find the patient unresponsive. There also was a report of a possible history of substance abuse. Upon EMS arrival, the patient was tachycardic in the 150s with a blood sugar of 254. His initial blood pressure was 76/52. EMS reported that the patient was combative and altered with a possible right-sided facial droop. They called for a medical command to administer 5 mg of IM midazolam to better control the patient so that treatment could be started. En route to the emergency department, he received 200 mL of intravenous normal saline solution. On arrival to the ED, the patient’s vitals were as follows: heart rate of 95 beats per minute, respiratory rate of 17 breaths per minute, blood pressure was 129/76 mmHg, 96.3 degrees F rectal temperature, 97% saturation in room air, and blood sugar of 309.

Given the facial droop and concern for stroke, a “Stroke Alert” was initiated. The doorway exam showed a subtle right-sided facial droop and left-sided weakness of the upper and lower extremities. Per protocol, he went directly for a noncontrast CT brain. The CT showed old small lacunar infarcts without definite acute findings. His physical exam found mild tachycardia, clear though shallow lungs, a non-distended soft abdomen without masses, and a lethargic sensorium that was resistant to care. His neurological exam was notable for PERRL and EOMI, no gag reflex, subtle right-sided nasolabial flattening, left upper extremity held in hemiplegic appearance with immediate drop to the bed, decreased spontaneous left leg motor, and a positive right Babinski reflex. Right-sided motor and all sensation were intact. Ocular confrontation did not suggest visual field cuts. He was mute and did not appear to understand or follow commands. His Glasgow Coma Scale was 9, and he had a NIH stroke scale of 20.

His last known well time was more than 6 hours prior to arrival in the ED. As such, he was not a candidate for acute thrombolysis. Further workup included a computed tomography angiography (CTA) of the head and neck which showed no large vessel occlusion, significant stenosis, or aneurysm. The CT also noted that the thoracic aorta and aortic arch vessels were patent and normal in caliber. Chest X-ray was unremarkable including normal mediastinal contours. Labs were notable for BUN 32 mg/dL, creatinine 6.2 mg/dL, blood sugar 309 mg/dL, lactic acid 5.6 mg/dL, CK 3985U/L, WBC 19 × 10^9^/L, and hemoglobin 9.7 g/L. Urinalysis showed cloudy urine with 2 + glucose, 3 + blood, 3 + protein, 6–15 WBCs, and cellular casts. PT/INR was 16.5/1.42 and PTT was 31.6. Toxicology was positive for benzodiazepines (administered by EMS).

The patient received 300 mg of rectal aspirin. Based on the above labs and imaging, the patient was admitted with the following diagnoses: acute stroke, acute kidney injury, lactic acidemia, rhabdomyolysis, severe sepsis, and UTI. At this point, the hospital received identification for the patient from a coworker. He was noted to be 58 years old with a history of hypertension.

Approximately 2.5 hours after the presentation, his blood pressure was noted to be 194/76, then 220/137. Fifteen minutes later, his blood pressure dropped to 83/64 without medication intervention. The patient’s abdomen was noted to be distended, and he remained hypotensive despite fluid resuscitation. A CT abdomen/pelvis with IV contrast was ordered which showed a large abdominal aortic aneurysm and large retroperitoneal hematoma, findings highly suspicious for a ruptured AAA. Emergent transfer to a higher medical center was arranged for endovascular surgery.

A repeat CTA of the chest/abdomen/pelvis at the receiving hospital showed a stable retroperitoneal hematoma and aneurysm that extended to the iliac arteries with extensive plaque calcification. The thoracic aorta was found to be normal in appearance. MRI brain showed a large area of diffusion restriction in the right occipital lobe favoring acute/subacute infarction with associated mass effect and partial effacement of the occipital horn of the right lateral ventricle. Additional scattered foci of diffusion restriction were favored to be embolic in etiology per the radiology report. There was a concern for the proximal embolic source (cardiac or aortic arch), but they were unable to identify the source of the embolic shower. Hypercoagulable studies were unrevealing. They intubated him after specialty evaluation. The endovascular infrarenal aorta to iliac bifurcation surgery went well. He received a tracheostomy and gastrostomy due to continued poor neurologic examinations. He was able to follow commands and mouth words. He remained with left-sided weakness. Bubble study echocardiogram was unremarkable except for LVH and trace multi-valve regurgitation. He had a normal ejection fraction. An MRI done 5 days after the presentation showed evidence of multiple diffuse strokes. He had acute/early infractions of the right occipital, and bilateral cerebellar and cerebral hemispheres. Radiologic impression favored embolic origin due to the diffuse bilateral involvement. Notably, this patient was noted to have some scattered atherosclerotic plaques in the aortic arch, but there was no evidence of aneurysm in the thorax. Additionally, there was no evidence of atrial fibrillation at any point during the patient encounter.

## Discussion

We present an unusual case that presented with acute neurologic deficits suggestive of a CVA but appears to have had a root cause of AAA rupture with showering of multiple emboli to the brain. The initial presentation of this patient was most concerning for acute CVA, and patients presenting with these symptoms require an initial stroke workup. Patients require immediate consideration for the use of thrombolytics and possible neurosurgical endovascular intervention. While the initial CT may not always show acute strokes, continued CVA workup including MRI and serial neurologic assessments are recommended for patients with focal neurologic deficits [4]. However, it is important to remember that acute neurologic findings may also be suggestive of other various pathologies [5], including AAA rupture.

The typical presentation for AAA rupture is severe abdominal and back pain [3, 6], but if a patient has become hemodynamically unstable, they may no longer be able to provide any history or symptoms and may present in severe shock. In this case, the patient may have experienced these symptoms. However, since he did not present to the emergency department coherent, we did not know how he was feeling prior to his acute deterioration and change in mental status. A key takeaway from this case is realizing that while the most common presentation of a life-threatening condition needs to be considered, it may not be the only possibility. This is especially true when a patient presents obtunded, adding many possible diagnoses to the differential.

The possibility of an acute CVA (or myocardial infarction and pericardial tamponade) can be associated with AAA rupture if it involves the carotid or coronary arteries. There may also be the appearance of peripheral upper extremity paresis and paresthesias if there is an interruption of the blood flow through the subclavian arteries. These conditions can have focal neurologic findings in the lower extremities if the blood flow to the spinal nerves is interrupted, or the blood flow to the extremities is compromised [7, 8]. As noted in prior literature, giving the standard of care thrombolytics for acute CVA can have a deleterious effect especially if there is an aortic dissection [9].

Additionally, when a “John Doe” presents to the emergency department, past medical history is unknown. While a patient may have a facial droop and focal weakness, those symptoms may not be new. While acute neurologic deficits require immediate attention, it is important to also consider diagnoses outside of neurologic disease, as weakness and facial asymmetry may be from old cerebral infarcts rather than an acute injury. Similarly, patients may have a history of known associated conditions, such as a dilated AAA, that might have led to consideration of ruptured AAA earlier in the clinical course had this patient’s history been known.

Furthermore, when a patient has one symptom or sign that does not fit a certain clinical diagnosis, it is important to properly evaluate that issue and not rule out a diagnosis based solely on one piece of clinical information. This patient went from hypotensive to hypertensive before hypotension again. An AAA rupture typically presents with hypotension [10]. However, posterior aortic wall rupture tends to present with less severe hypotension than anterior wall rupture [11]. This is presumably due to a tamponade effect in the retroperitoneum. A possible posterior rupture combined with a history of hypertension and acute onset of pain and discomfort may lead to normotensive or hypertensive presentations in ruptured AAA.

## Conclusions

In conclusion, ruptured AAA typically presents with abdominal pain, shock, and a pulsatile mass but can occasionally present with focal neurologic deficits, hypertension, and AMS. While AMS and unilateral neurologic findings may be most commonly seen in patients after a CVA, repeat examinations and continual vital signs are essential in identifying other potential causes of neurologic deficits, including AAA rupture.

## Data Availability

Data sharing is not applicable to this article as no datasets were generated or analyzed during the current study.

## Abbreviations

AAA: Abdominal aortic aneurysms
CVA: Cerebrovascular accident
AMS: Altered mental status
EMS: Emergency medical services
CTA: Computed tomography angiography
